# GLAST Deficiency in Mice Exacerbates Gap Detection Deficits in a Model of Salicylate-Induced Tinnitus

**DOI:** 10.3389/fnbeh.2016.00158

**Published:** 2016-08-17

**Authors:** Hong Yu, Kim Vikhe Patil, Chul Han, Brian Fabella, Barbara Canlon, Shinichi Someya, Christopher R. Cederroth

**Affiliations:** ^1^Laboratory of Experimental Audiology, Department of Physiology and Pharmacology, Karolinska InstitutetStockholm, Sweden; ^2^Department of Otolaryngology, Head and Neck Surgery, First Hospital of JiLin UniversityChangchun, China; ^3^Department of Aging and Geriatric Research, University of FloridaGainesville, FL, USA; ^4^Howard Hughes Medical Institute and Laboratory of Sensory Neuroscience, The Rockefeller UniversityNew York, NY, USA

**Keywords:** hearing loss, salicylate, tinnitus, gap detection, pre-pulse inhibition, startle response, mouse, disease models

## Abstract

Gap detection or gap pre-pulse inhibition of the acoustic startle (GPIAS) has been successfully used in rat and guinea pig models of tinnitus, yet this system has been proven to have low efficacy in CBA mice, with low basal GPIAS and subtle tinnitus-like effects. Here, we tested five mouse strains (CBA, BalbC, CD-1, C57BL/6 and 129sv) for pre-pulse inhibition (PPI) and gap detection with varying interstimulus intervals (ISI) and found that mice from a CBA genetic background had the poorest capacities of suppressing the startle response in the presence of a pre-pulse or a gap. CD-1 mice displayed variable responses throughout all ISI. Interestingly, C57BL/6, 129sv and BalbC showed efficient suppression with either pre-pulses or gaps with shorter ISI. The glutamate aspartate transporter (GLAST) is expressed in support cells from the cochlea and buffers the excess of glutamate. We hypothesized that loss of GLAST function could sensitize the ear to tinnitus-inducing agents, such as salicylate. Using shorter ISI to obtain a greater dynamic range to assess tinnitus-like effects, we found that disruption of gap detection by salicylate was exacerbated across various intensities of a 32-kHz narrow band noise gap carrier in GLAST knockout (KO) mice when compared to their wild-type (WT) littermates. Auditory brainstem responses (ABR) and distortion-product otoacoustic emission (DPOAE) were performed to evaluate the effects on hearing functions. Salicylate caused greater auditory threshold shifts (near 15 dB) in GLAST KO mice than in WT mice across all tested frequencies, despite similarly reduced DPOAE. Despite these changes, inhibition using broad-band gap carriers and 32 kHz pre-pulses were not affected. Our study suggests that GLAST deficiency could become a useful experimental model to decipher the mechanisms underlying drug-induced tinnitus. Future studies addressing the neurological correlates of tinnitus in this model could provide additional insights into the mechanisms of tinnitus.

## Introduction

Tinnitus remains an untreatable condition frequently associated with stress, anxiety or depression (Baguley et al., 2013), and affects 10–15% of the population. In spite of increasing attention towards the understanding and treatment of tinnitus, experimental efforts in the field remain relatively limited in comparison to the numerous clinical reports (Cederroth et al., 2013). Experimentally, the induction of tinnitus is achieved through noise overexposure or the administration of ototoxic drugs (e.g., salicylate, quinine or cisplatin; Stolzberg et al., 2012; von der Behrens, 2014).

Advances in the field of tinnitus have been made due to the development of behavioral methods to objectively assess the perception of non-existing sounds. Gap pre-pulse inhibition of the acoustic startle reflex (GPIAS), validated in a rat model of tinnitus using the operant conditioning paradigm (Turner et al., 2006; Turner and Parrish, 2008), resembles pre-pulse inhibition of the acoustic startle reflex (PPI or PPIAS), whereby a reduction in the response to an intense stimulus is observed when preceded by a subthreshold intensity pre-pulse (Ison et al., 1973; Graham, 1975). Animal models or humans with schizophrenia or bipolarity disorders have deficits in inhibiting the startle reflex with a pre-pulse as a consequence of a dysfunction in the sensorimotor gating mechanism (Braff et al., 1978; DelPezzo and Hoffman, 1980). In contrast to PPI, which uses a low-intensity pre-pulse to inhibit the startle reflex, GPIAS presents a silent gap embedded in a continuous carrier noise. Despite startle suppression being calculated similarly for both GPIAS and PPI, these use different neural pathways to regulate inhibition. Lesion studies have shown that the auditory cortex regulates GPIAS but not PPI (Bowen et al., 2003). When used in the context of tinnitus, as an animal’s tinnitus closely matches the background noise, the startle reflex is less suppressed by the pre-pulse gap because tinnitus interferes with the optimal inhibition of the startle reflex mediated by the gap. As a consequence, affected animals display greater startle response (meaning less inhibited) than in the absence of tinnitus. The use of GPIAS for the assessment of tinnitus has been supported by additional neuronal correlates of tinnitus such as increased spontaneous firing rates in the dorsal cochlear nucleus (DCN; Li et al., 2013), hyperactivity in the inferior colliculus (Holt et al., 2010) and remapping of the auditory cortex (Engineer et al., 2011).

CBA mice have been conventionally used in auditory research for their excellent hearing abilities. In contrast, C57BL/6, in which mutant strains have been traditionally developed, display age-related hearing loss due to a point mutation on the *Cdh23* gene (Ohlemiller and Gagnon, 2004) and thus have been less accepted in the auditory field. However, in the absence of tinnitus, suppression of the startle response with the presence of a gap in narrow band carriers remains highly inefficient in CBA mice (10–50%; Middleton et al., 2011; Llano et al., 2012; Hickox and Liberman, 2014) when compared to that in rats (40–60%; Turner et al., 2006; Turner and Parrish, 2008; Engineer et al., 2011; Su et al., 2012; Yi et al., 2016), consequently offering a small dynamic range to distinguish tinnitus-like effects. Thus, improving the basal suppression of gap detection in mice would increase the confidence window for detecting tinnitus. Curiously, mice appear to be resistant to developing tinnitus, as only 20–50% of the mice that are exposed to noise display behavioral evidence of tinnitus (Middleton et al., 2011; Li et al., 2013), compared to 70–75% of that in rats (Wang et al., 2009; Zhang et al., 2011; Ruttiger et al., 2013). These observations are consistent with the notion that mice are more resilient than guinea pigs to drug-induced hearing loss by, for instance, aminoglycoside antibiotics or the anti-cancer drug cisplatin (Poirrier et al., 2010). Overall, a smaller number of mice display noise-induced changes in GPIAS than do rats, and this species appears more resistant to drug-induced hearing loss than are guinea pigs.

The glutamate aspartate transporter (GLAST) appears as a potential candidate to explain such differences. GLAST belongs to the family of glutamate transporters that stabilize the extracellular environment and maintain cell-to-cell communication. GLAST, but not GLT1, has been identified in the hearing organ, the cochlea (Jin et al., 2003). GLAST is present in the inner phalangeal cells (IPCs) that surround the sensory inner hair cells (IHCs) and afferent neuron synapse (Ruel et al., 2007), where it pumps back excessive glutamate released by sensory cells (Glowatzki et al., 2006) during noise exposure (Hakuba et al., 2000; Chen et al., 2010). As a consequence, mice lacking GLAST show greater hearing threshold shifts and synaptic damage than do wild-type (WT) mice after noise exposure (Hakuba et al., 2000; Chen et al., 2010). GLAST is hardly detectable in the cochlea of rats or guinea pigs and is abundant in the mouse cochlea (Jin et al., 2003). This is why we hypothesized that GLAST abundance in the murine cochlea could underlie the resilience of mice to inner ear insults by drugs, and potentially tinnitus.

Research on tinnitus would benefit from the use of mice, whose species offers facilitated genetic manipulations for understanding the mechanisms that are related to this auditory disorder. In the present study, we address the limitations of using mice by: (i) increasing the dynamic range of startle suppression in the presence of a gap; and (ii) identifying a protein whose disruption exacerbates gap detection deficits induced by salicylate. By combining these two advances, we propose a new mouse model for investigating tinnitus.

## Materials and Methods

### Experimental Animals

Experimental procedures on animals performed at the Karolinska Institutet were in accordance with the guidelines and regulations set out by the University and *Stockholm’s Norra Djurförsöksetiska Nämnd*. Experiments performed at the Rockefeller University were approved by the Institutional Animal Care and Use Committee of The Rockefeller University. GLAST knockout (KO) mice on a C57BL/6 background (Watase et al., 1998) and the other listed strains (from Charles Rivers) were maintained at 19–21°C in a 50%–50% light-dark cycle.

GLAST KO mice and their WT littermates were obtained from heterozygous crosses. We observed a non-mendelian distribution in the progeny of crosses between heterozygous KO mice (*n* = 145): 28% WT animals, 61% heterozygous KO animals, and only 10% homozygous KO animals. When crossing male homozygotes with female heterozygotes, we obtained 80% heterozygous and 20% homozygous KO animals among the progeny. In addition, homozygous females appeared highly anxious and displayed pup-killing behavior. Only male mice between 2 and 4 months of age were used in this study. Baseline auditory thresholds of GLAST KO mice measured by auditory brainstem responses (ABR) within this age range were similar to those of WT littermates (8 kHz = 35.71 ± 2; 16 kHz = 19.29 ± 0.7; 32 kHz = 29.64 ± 1.7; KO: 8 kHz = 38 ± 1.7; 16 kHz = 21 ± 1; 32 kHz = 32 ± 1.33; *p* = 0.97 by a 2-way ANOVA).

The animals had free access to water and food. They were injected daily with intraperitoneal (i.p.) sodium salicylate (Sigma, S3007) at 300 mg/kg diluted in 0.9% NaCl for three consecutive days. Hearing threshold measures and behavior tests were performed 2 h after the last injection, as previously described (Guitton et al., 2003).

### Auditory Brainstem Responses

Mice were anesthetized with 80 mg/kg ketamine and 12 mg/kg xylazine for measurements of ABR. The positive needle electrode was inserted subdermally at the vertex, the negative electrode was placed beneath the pinna of the left ear, and the ground electrode was located near the tail. ABR were evoked by tone bursts of 8, 16 and 32 kHz, produced by a closed-field electrostatic speaker connected to a driver (EC-1 and ED-1, Tucker-Davis Technologies). The 5-ms signals were presented 33.3 times per second; their 0.5-ms onsets and offsets were tapered with a squared cosine function. The speaker’s audio output was transmitted into the ear through a custom acoustic coupler. Sound pressure levels were measured with a calibrated microphone and preamplifier, connected to a conditioning amplifier (4939-A-011 and 2690-A-0S1, Brüel and Kjær). The response was amplified 10,000× and bandpass filtered at 0.3–3 kHz (P55, Natus Neurology Inc.). The amplified response was then digitally sampled at 10-μs intervals with a data acquisition device (USB-6210, National Instruments), controlled by custom software (LabVIEW 2010, National Instruments). The responses to 1000 bursts were averaged at each intensity level to determine the threshold; the threshold is defined as the lowest level at which a response peak is distinctly and reproducibly present. For each frequency, the sound pressure level was decreased from 100 dB SPL in 5 dB steps, until the threshold was reached and confirmed with one replicate measure. Threshold shifts were measured against individual’s baseline values.

### Distortion-Product Otoacoustic Emissions

Distortion-product otoacoustic emissions were elicited with an acoustic assembly, consisting of two electrostatic speakers (EC-1, Tucker-Davis Technologies), to generate primary tones and a miniature microphone (EK-23103, Knowles) to measure ear-canal sound pressure. The speakers and the microphone were both calibrated with the calibration microphone described above. The 2*f*_1_ − *f*_2_ distortion product was measured with *f*_1_ = 6–24 kHz, *f*_2_/*f*_1_ = 1.25 and the stimulus levels *L*_1_ = *L*_2_ = 75 dB SPL. The acoustic signal was amplified by a preamplifier (ER-10B+, Etymotic Research), and the sound pressure measured in the ear canal was digitally sampled at 10-μs intervals with the data acquisition system described above. Each frequency pair was presented for 1 s. After computing fast Fourier transforms and averaging them over 10 consecutive traces, we determined for each frequency pair the amplitudes of the 2*f*_1_ − *f*_2_ distortion product and of the noise floor measured at ±100 Hz from 2*f*_1_ − *f*_2_; this procedure required 17 s of data acquisition and processing.

### Testing of the Interstimulus Interval

Interstimulus intervals (ISI) were tested using the SR-Lab startle response system from San Diego Instruments as previously described with minor modifications (Lowry et al., 2013). Animals were acclimated to the procedure on day 1, and were tested for PPI on day 2 and GPIAS on day 5. Background sound level (unfiltered white noise) was 65 dB SPL for PPI sessions, with pre-pulses of 75 dB SPL. For GPIAS, carrier level was 80 dB SPL and silent gaps (absence of stimulus) went down to the noise floor of the SR-Lab chamber. Startle pulses were presented at 115 dB SPL. Trials varied pseudo-randomly in their ISI by 10 ms, from 150 to 0 ms. Ten trials per ISI were tested. Inter-trial time interval varied randomly from 8 to 15 ms. Twenty startle-only trials were presented before the session, and five startle-only trials at the end in order to assess habituation. One whole session of PPI or GPIAS lasted 1 h.

### Tinnitus Evaluation by the Gap Detection Method

To test frequency-specific gap detection deficits, we developed a custom-made set-up. Tests were performed in a sound-attenuating chamber (ENV-022S, Med Associates, Inc.). whose noise floor was 55 ± 0.5 dB SPL. In the case of PPI tests, which were used to verify the normal sensorimotor gating, white noise was generated by a function generator (DS340, Stanford Research Systems) and filtered (Wavetek 852 Dual HI/LO Variable Analog Filter, Butterworth filtered, 48 dB/octave roll-off) to 1 kHz-wide narrowband noise centered at a given frequency or broadband noise (BBN) and presented at a given sound intensity (60, 65, 70, 75, 80 dB SPL) in a silent environment for a duration of 50 ms through a speaker (NX-6, Power Acoustik) positioned in front of the animal. To startle an animal, a 20-ms white noise startle pulse of 115 dB SPL was delivered from a second speaker positioned above the animal, 70 ms after the pre-pulse. Calibration was done using the microphone, preamplifier and conditioning amplifier mentioned above.

For the gap detection tests that were used to evaluate tinnitus perception, the same set-up was used. The gap carrier was filtered as above into a broadband or 1 kHz-wide narrowband noise centered at a given frequency and presented at a given sound intensity (60, 65, 70, 75, 80 dB SPL). A relay switch was used to silence the noise for 50 ms, a gap with 0.1 ms rise and fall times (RT/FT), which was followed 15 ms later by a 20-ms white noise startle pulse of 115 dB SPL. The startle response was captured through an electromagnetic coil, bandpass filtered at 3–100 Hz and amplified 10× (P55, Natus Neurology Inc.). Gap detection or PPI was quantified as the percentage decrease in the peak amplitude of the startle response, when a warning gap or pre-pulse preceded the startling noise in comparison to the amplitude when no gap or pre-pulse was present [(1 − the ratio) × 100] (Engineer et al., 2011), using a similar representation as used in PPI studies (greater suppression of the startle reflex closer to 100%). The more tinnitus fills the gap, the less inhibition of the startle is observed. Only naïve animals were used in this study.

#### Experimental Procedure for the Screening of the Putative Tinnitus Frequency

This procedure was performed on a small group of animals (*n* = 3) to identify the putative tinnitus frequency at which a deeper analysis could be performed with a greater number of animals (see below). Here, PPI was not performed in order to avoid excessive habituation to the startle stimulus.

In order to acclimatize the animals to the testing procedure, a 30-min preliminary test was performed on day 1. On the following day (day 2), the experiment comprised a session of three consecutive blocks.

##### Block 1

The first block started with a 5-min acclimatization to silence, followed by 20 startle pulses; each of the pulses was 20 ms in duration and at 115 dB SPL. The time between each trial was random and varied between 8 and 12 s.

##### Block 2

The second block consisted in testing gap detection at various carrier frequencies (BBN, 8, 10, 12, 16, 20, 24, 32-kHz) of 80 dB SPL with 20 trials per frequency, with or without silent gaps (50 ms). Each frequency was tested sequentially with 20 trials. Each trial was performed with 20-ms startle pulses and an ISI of 15 ms.

##### Block 3

The final block comprised five trials only with startle pulses to be compared with those of the first block to assess habituation. A total of 185 trials was presented in approximately 40 min.

One day after the testing (day 3), animals were treated with salicylate daily for 2 days and tested on the third day of administration (day 5) with the same procedure.

#### Experimental Procedure for the Validation of the Putative Tinnitus Frequency

This procedure was performed to validate the findings from the frequency screening performed above. In order to acclimatize the animals to the testing procedure, a 30-min preliminary test was performed on day 1. On the following day (day 2), the experiment comprised a session of four consecutive blocks.

##### Block 1

The first block started with a 5-min acclimation to silence, followed by 20 startle pulses; each of the pulses was 20 ms in duration and at 115 dB SPL. The time between each trial was random and between 8 and 12 s.

##### Block 2

A second block of stimuli consisted in testing PPI with 20 trials with or without pre-pulses (50 ms), first consisting of a BBN of 75 dB SPL, and then consisting of a 32 kHz centered narrowband noise of successive intensities of 60, 65, 70, 75 and 80 dB SPL in a quiet background. Each trial was performed with 20-ms startle pulses and an ISI of 70 ms.

##### Block 3

The third block consisted in testing gap detection with 20 trials, with or without silent gaps (50 ms), first embedded in a broadband carrier noise at 75 dB SPL and then embedded in a 32-kHz centered narrowband noise of successive intensities of 60, 65, 70, 75 and 80 dB SPL. Each trial was performed with 20-ms startle pulses and an ISI of 15 ms.

##### Block 4

The final block comprised five trials only with startle pulses to be compared with those of the first block to assess habituation. A total of 265 trials were presented in approximately 55 min.

One day after the testing (day 3), animals were treated with salicylate daily for 2 days and tested on the third day of administration (day 5) with the same procedure.

### Quantitative Real Time-PCR

SybrGreen qRT-PCR assays from cochlear extracts were performed as previously described (Meltser et al., 2014; Vikhe Patil et al., 2015). A mean quantity was calculated from triplicate PCR for each sample, and this quantity was normalized with the geometric mean of the three most stable genes out of six reference genes (tubulin β, Tubb; glyceraldehyde-3-phosphate dehydrogenase, G3pdh; transferring receptor 1, Trf1R; Tubulin α2, Tuba2; hypoxanthine phosphoribosyltransferase, HPRT; and Cyclophilin B) selected using the geNorm algorithm as described (Vandesompele et al., 2002). Normalized quantities were averaged for three technical replicates for each data point and represented as mean ± SD. The highest normalized relative quantity was arbitrarily designated 1.0. Fold changes were calculated from the quotient of means of these normalized quantities and reported as ± SEM. The primers for *eaat1* used are F: 5′-GGGAAGATGGGGATGCGAG-3′ and R: 5′-GCCGAAGCACATGGAGAAG-3′.

### Statistical Analysis

Two-way ANOVA and a Bonferroni *post hoc* were used for statistical analysis (Prism version 4.0, GraphPad software). Differences were considered significant if *P* < 0.05. Animals that failed to respond to the startle (any peak-to-peak response above noise floor was considered a startle) or failed to inhibit the startle in the presence of a pre-pulse before salicylate treatment (any decrease in startle amplitude during pre-pulse trials vs. startle only trials) were excluded from the analysis (near 5%). When performing PPI tests using a pre-pulse of 80 dB SPL, nearly 10% of the pre-pulses elicited a startle, which then completely suppressed the startle response. The greater the intensity of the pre-pulse, more efficient was the inhibition of the startle response. As a consequence, trials in which the 80 dB pre-pulse induced a startle response before the startle pulse (10% of the 80 dB pre-pulse trials) were excluded from the analysis.

## Results

### Shorter Interstimulus Interval Improves GPIAS in Specific Mouse Strains

**Figure 1:**
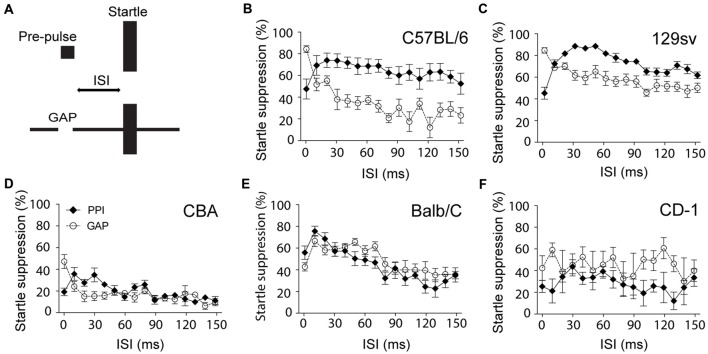
**Shorter interstimulus intervals (ISI) improves gap-mediated suppression of the startle response. (A)** Schematic model of the experiment. Male C57BL/6 **(B)**, 129sv **(C)**, CBA **(D)**, Balb/C **(E)** and CD-1 **(F)** male mice (2–4 months old) were exposed to pre-pulse inhibition of the acoustic startle reflex (PPIAS) (black diamonds) and gap pre-pulse inhibition (PPI) of the acoustic startle (GPIAS; open circles) sequentially. ISI was modified by steps of 10 ms. Average startle amplitudes before and after each paradigm were not affected. Data represent means ± SEM (*n* = 8).

When developing a custom-made gap detection set-up, we found that the ability of C57BL/6 mice to inhibit the startle response in the presence of a gap was almost null when using similar settings to those used for PPI (duration of the pre-pulse: 50 ms; ISI: 70 ms; startle duration: 20 ms; startle intensity: 115 dB). This particular paradigm only reduced the startle response by 20% (data not shown). We found that GPIAS was particularly sensitive to modifications of the ISI. We observed that in both C57BL/6 and 129sv mice, unlike for PPI, the shorter the ISI, the greater the inhibition of startle response, achieving up to 80% in C57BL/6 and 129sv mice (Figures 1B,C). Measures of the startle amplitude before and after each PPIAS or GPIAS session confirmed the lack of habituation during this test (data not shown). While this pattern was present in CBA mice, it did not exceed more than 50% at the lowest ISI, strongly suggesting that the inhibition of the startle reflex in this strain is not efficient (Figure 1D). This trend was not observed when using other genetic backgrounds such as CD-1 and Balb-C mice (Figures 1E,F). In Balb-C, PPI and GPIAS followed a similar course over varying ISIs, and CD-1 had highly variable responses, suggesting they would require additional acclimatization sessions (as typically performed with rats) to obtain a more robust reflex response. Overall, it is concluded that ISI is a critical parameter for improving gap detection in C57BL/6 and 129sv mice, and that shorter ISIs can be used to increase the basal level of suppression of the startle response in these strains in order to provide greater dynamic range to detect deficits in gap detection.

### Salicylate Causes Severe Gap Detection Deficits in GLAST KO Mice

The basal level of startle suppression in the presence of a gap was further improved using Longenecker and Galazyuk (2012) recommendations by: (i) adjusting speaker non-linearity by calibrating the intensity output for each filtered narrowband noise; (ii) suppressing echo and reverberation by covering the interior walls with sound-insulating foam; and (iii) replacing the acrylic animal restrainer with a non-resonating perforated plastic pipette box. Because noise is effective in inducing tinnitus in nearly 50% of animals, we used salicylate, which is the most commonly used drug in animal models of tinnitus (Cazals, 2000; von der Behrens, 2014). Salicylate has an advantage over noise in that it has previously been used in humans to induce tinnitus (3.9 g salicylate/day for 5 days; Mongan et al., 1973; McFadden et al., 1984).

We screened various 1-kHz narrowband frequencies (from 8 to 32 kHz) in a small group of animals (*n* = 3) to identify the putative tinnitus frequency, meaning the frequency at which GPIAS would be most affected by salicylate treatment according to a previous model (300 mg/kg/day for 3 days, Guitton et al., 2003). We used a carrier noise of high intensity (80 dB SPL) to maximize the inhibition of the startle by the gap and identify the frequencies with the greatest changes. This initial screening showed that both WT and KO mice treated with salicylate exhibit greater deficits in sensing the gap at 32 kHz (two-way ANOVA, Genotype and Treatment Factor: *F*_(7,64)_ = 6.108, *p* < 0.0001; Frequency Factor: *F*_(3,64)_ = 11.44, *p* < 0.0001, data not shown).

**Figure 2:**
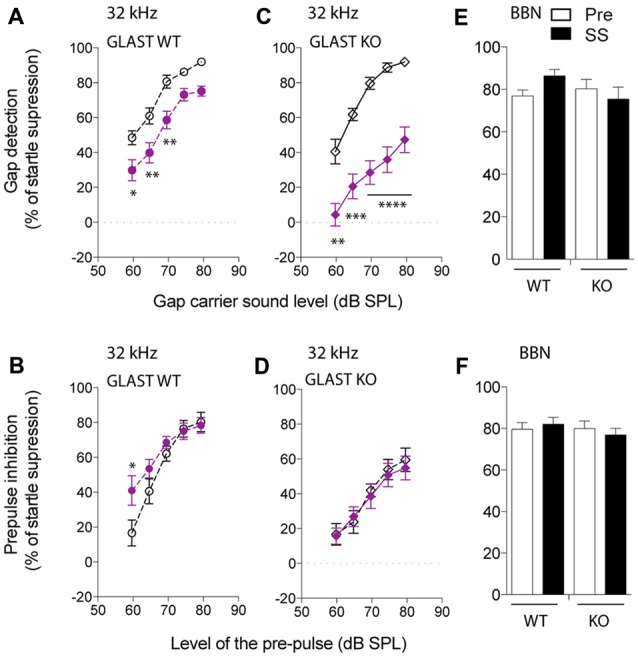
**Glutamate aspartate transporter (GLAST) deficiency facilitates the induction of tinnitus by salicylate.** GPIAS in wild-type (WT) **(A)** and GLAST knockout (KO) **(C)** mice before (dark) and 2 h after the last salicylate (SS) administration (purple) using a 32-kHz narrowband carrier noise presented with a 5 dB step increase in intensity. PPIAS in WT **(B)** and GLAST KO **(D)** mice before (dark) and after (purple) salicylate treatment using a 32-kHz narrowband pre-pulse presented with a 5 dB step increase in intensity. Normal gap **(E)** and pre-pulse sensing **(F)** in WT and GLAST KO mice before (white) and after (black) the injection of SS using a broadband noise (BBN) at 75 dB SPL carrying a silent gap (GPIAS session) or a 75 dB SPL broadband pre-pulse presented in a silent environment (PPI session). Pre-treatment values are shown in dark, and SS values are shown in filled symbols in purple. WT mice are shown with circles and GLAST KO mice are shown with diamonds. Data represent means ± SEM (*n* = 8–14). *P < 0.05, **P < 0.01, ***P < 0.001 and ****P < 0.0001, by a two-way ANOVA with *post hoc* Bonferroni test.

We next focused on the 32-kHz narrowband noise to perform both GPIAS, using different carrier intensities, and PPI, using increasing pre-pulse intensities. PPI was used as a control for normal temporal processing or sensory motor gating. Before salicylate administration, WT animals increasingly detected the gap with increasing intensities of a narrowband carrier noise centered at 32 kHz, inhibiting their startle reflexes up to 91% (Figure 2A). After 3 days of salicylate administration, the ability of WT mice to repress their startle reflexes decreased by 25% at all carrier intensities tested (*p* < 0.0001 by a two-way ANOVA with Bonferroni *post hoc* test, Treatment Factor, *F*_(1,110)_ = 40.46, *p* < 0.0001; Carrier Intensity Factor, *F*_(4,110)_ = 35.36, *p* < 0.0001; Figure 2A). Salicylate did not affect PPI with the exception of the lowest intensity of pre-pulse tested, suggestive of hyperacusis (*p* = 0.03 by a two-way ANOVA with Bonferroni *post hoc* test, Treatment Factor, *F*_(1,111)_ = 4.64, *p* = 0.0333; Pre-pulse Intensity Factor, *F*_(4,111)_ = 25.40, *p* < 0.0001; Figure 2B). The overall gap detection deficits, although significant, appeared to be relatively small.

Before salicylate treatment, GLAST KO mice displayed GPIAS as efficiently as WT mice (two-way ANOVA, Genotype Factor: *F*_(1,79)_ = 0.4351, *p* = 0.5114; Carrier Intensity Factor: *F*_(4,79)_ = 57.0, *p* < 0.0001). However, after salicylate treatment, the ability of GLAST KO mice to detect the gap was severely impaired throughout all carrier intensities tested (two-way ANOVA with Bonferroni *post hoc* test, Treatment Factor: *F*_(1,89)_ = 117.5, *p* < 0.0001; Carrier Intensity Factor: *F*_(4,89)_ = 16.02, *p* < 0.0001; Figure 2C). Although PPIAS at 32 kHz appeared to be lower in GLAST KO mice than in WT mice (two-way ANOVA, Genotype Factor: *F*_(1,105)_ = 17.19, *p* < 0.0001; Pre-pulse Intensity Factor: *F*_(4,105)_ = 27.54, *p* < 0.0001), it was not affected by salicylate administration (two-way ANOVA, Treatment Factor: *F*_(1,105)_ = 0.2299, *p* = 0.6326; Pre-pulse Intensity Factor: *F*_(4,105)_ = 15.72, *p* < 0.0001, Figure 2D), suggesting that the disruption of gap detection is more likely a tinnitus effect rather than a deficit in temporal processing or in auditory sensitivity (hyperacusis-like phenomenon).

To confirm the frequency-specific effects on GPIAS observed after the treatment with salicylate, we used a BBN Gap or pre-pulse (BBN PPI) at 75 dB SPL, and next verified whether inhibition of the startle response was affected before and after salicylate administration in both WT and GLAST KO mice. GPIAS in a BBN carrier was equally efficient before or after salicylate treatment in both genotypes (two-way ANOVA, Genotype Factor: *F*_(1,40)_ = 0.7098, *p* = 0.4045; Treatment Factor: *F*_(1,40)_ = 0.2667, *p* = 0.6084, Figure 2E). Similarly, the efficacy of BBN PPI, which is also used to assess normal temporal processing (Turner et al., 2006; Turner and Parrish, 2008; Middleton et al., 2011; Llano et al., 2012), was identical in WT and mutant animals both before and after the administration of salicylate (two-way ANOVA, Genotype Factor: *F*_(1,46)_ = 0.4673, *p* = 0.4976; Treatment Factor: *F*_(1,46)_ = 0.009994, *p* = 0.9208, Figure 2F). These control experiments confirmed that BBN PPIAS and GPIAS are not affected by salicylate in both WT and KO mice and support the notion that salicylate causes greater gap detection deficits at 32 kHz in GLAST KO than it does in WT mice.

**Figure 3:**
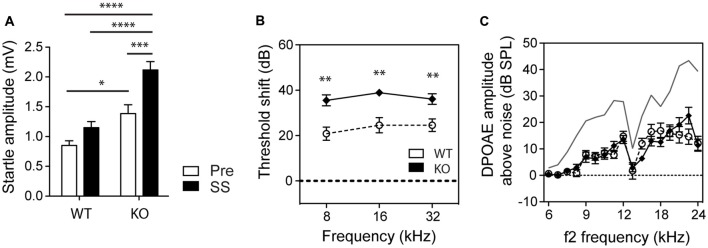
**GLAST deficiency sensitizes mice to salicylate-induced hearing loss but without affecting outer hair cell function. (A)** GLAST KO mice subjected to startle pulses showed significantly larger startle responses than did WT mice. Salicylate (black, SS) enhanced the startle reflexes of GLAST KO mice. Pre-treatment values are shown in white. Data represent means ± SEM (*n* = 8–14). Auditory threshold shifts **(B)** and distortion-product otoacoustic emissions (DPOAEs **C)** of GLAST WT (white circles) and homozygous (black diamonds) mice with sodium salicylate at 300 mg/kg/day for 3 days, measured 2 h after the last injection. The uppermost curve (in gray) represents the mean for saline-treated WT control animals analyzed on the same day. Data of the auditory measures represent means ± SEM (*n* = 10–16). *P < 0.05, **P < 0.01, ***P < 0.001 and ****P < 0.0001, by a two-way ANOVA with *post hoc* Bonferroni test.

We also assessed how basal startle amplitudes were affected in WT and GLAST KO mice by salicylate. Salicylate treatment increased the startle amplitude in response to startle pulses alone in KO mice (two-way ANOVA, Genotype Factor: *F*_(1,56)_ = 37.90, *p* < 0.0001; Treatment Factor: *F*_(1,56)_ = 17.86, *p* < 0.0001, Figure 3A). Changes in hearing thresholds could account for differences in gap detection as well as in PPI. It is worth noting that salicylate caused a loss of hearing threshold by 20 dB across all frequencies (from 8 to 32 kHz) in WT mice and 35 dB in GLAST KO mice (two-way ANOVA, Genotype Factor, *F*_(1,57)_ = 35.03, *p* < 0.0001; Frequency Factor, *F*_(2,57)_ = 0.8140, *p* = 0.4482, *n* = 10–16, Figure 3B) with distortion-product otoacoustic emissions (DPOAEs) reduced by half, and in a similar way in WT and KO mice (Genotype Factor, *F*_(16,340)_ = 19.05, *p* = 0.9188; Frequency Factor, *F*_(16,340)_ = 1.248, *p* < 0.0001, Figure 3C). However, DPOAE measures were only performed at suprathreshold levels, and potential differences at threshold could have occurred. Still, the lack of differences in DPOAEs between WT and KO mice suggests that GLAST does not regulate outer hair cell function; rather GLAST could potentiate the effects of salicylate at the afferent synapse as suggested by the greater threshold shifts in GLAST KO mice. These findings are in agreement with previous work showing that salicylate potentiates glutamate-evoked responses in spiral ganglion neurons *ex vivo* (Ruel et al., 2008).

Such differences in salicylate-induced threshold shifts between WT and KO should have equally affected the perception of the pre-pulse or the perception of the gap and subsequent PPIAS and GPIAS. However, the findings indicate that this is not the case since PPI remained completely unaffected by salicylate treatment at 32 kHz where gap detection deficits were the greatest (Figures 2B,D). Second, we observed gap sensing deficits in GLAST WT and KO mice at 32 kHz but not when using a BBN as a carrier (Figure 2E), although similar hearing threshold shifts have been found across all frequencies tested (Figure 3B). Overall, our results suggest that the gap detection deficits observed here are reminiscent of tinnitus perception in the high-frequency area and are not due to hearing loss or defective temporal processing.

## Discussion

The present study shows that suppression of the startle response in the presence of a gap can be improved in C57BL/6 and 129sv mice by shortening the ISI and that constitutive loss of glutamate transporter function likely exacerbates the tinnitus-inducing effects of salicylate.

In some strains of mice, the efficacy of the startle suppression by the gap requires shorter ISI than with PPI. With a 15-ms ISI, gap detection suppressed the startle response by nearly 80% in C57BL/6 mice. Our findings contrast with those in the previous studies in rats by Ison and Bowen (2000), in which a biphasic response in the ability of the gap to suppress the startle was observed when varying the ISI . It is likely that these differences are related to species differences. Our results also underline the importance of the genetic background when performing GPIAS experiments. Previous studies have shown that PPI is highly influenced by genetic background and that the C57BL/6 strain displays rather efficient abilities to suppress the startle response in the presence of a pre-pulse (Willott et al., 2003). It would thus be interesting to evaluate how GPIAS is affected by varying ISIs in humans and how genetic background (e.g., different ethnicities) affects the efficacy of GPIAS.

In spite of the successful use of gap detection to identify physiological and molecular pathways involved in tinnitus (Engineer et al., 2011; Li et al., 2013, 2015; Kalappa et al., 2014, 2015), there has been a lot of debate regarding the use of gap detection for tinnitus evaluation (Campolo et al., 2013; Fournier and Hébert, 2013; Boyen et al., 2015; Galazyuk and Hébert, 2015). First, the low level of startle suppression in the presence of a gap reported in tinnitus studies that have used CBA mice leaves little margin to identify gap detection deficits and infer the presence of tinnitus (Longenecker and Galazyuk, 2011; Hickox and Liberman, 2014; Longenecker et al., 2014). Methods to discriminate between tinnitus and non-tinnitus animals have proven useful (Li et al., 2013, 2015) but an increase in the basal suppression level of the startle response offers a greater dynamic range for identifying tinnitus with greater confidence. Second, Hickox and Liberman (2014) performed a tinnitus study in which they adjusted the gap such that some of the trials were done with a gap closer to the startle stimulus (ISI = 0 ms) and some were done with a gap presented at greater lead times (ISI = 70 ms). Consistent with our findings (Figure 1), differences in gap detection between the two ISI conditions were found (being less efficient with greater lead times), however the authors interpreted that tinnitus “was not filling” the gap, since deficits in GPIAS were not observed at both ISIs (Hickox and Liberman, 2014). We believe the experimental conditions used were not optimal for inferring the presence of tinnitus as they used CBA mice, whose basal level of startle suppression in the presence of the gap was inefficient and even more variable with greater ISI. In addition, it has been shown that scopolamine, a muscarinic receptor blocker that disrupts cholinergic function in the brain, affects gap detection when the ISI is larger and not when the gap is closer to the startle stimulus (Ison and Bowen, 2000), meaning that different mechanisms operate GPIAS depending on the ISI. As a consequence, since GPIAS is a reflex response that relies on temporal processing and not on a conscious percept (meaning tinnitus does not fill the gap but interferes with the reflex response *per se*), gap detection deficits caused by tinnitus cannot be expected to be equally efficient at various lead times. The mechanism with which salicylate interferes solely with GPIAS and not PPI is not well understood, however it has been shown that gap detection operates in the auditory cortex, which is not the case of PPI (Bowen et al., 2003). Recent optogenetic studies in mice have shown that cortical inhibition is important in controlling perceptual gap detection (Weible et al., 2014). Since salicylate has been shown to alter the activity in the auditory cortex (Wang et al., 2006; Sun et al., 2009), we postulate that salicylate alters perceptual gap detection at the level of the auditory cortex. Additional behavioral methods in mice would be needed to confirm that the GPIAS deficits observed here correlate with a tinnitus percept.

Previous studies have shown that the ability to suppress the startle response in the presence of a gap improves with experience or with repeated pre-acclimatization sessions (Crofton et al., 1990; Ison and Bowen, 2000), and can reach 50% of startle suppression in CBA mice (Ison et al., 2002). The same applies to PPI (Plappert et al., 2006). We believe that the CBA strain, which is commonly used in the auditory field because of the well-preserved hearing, is not appropriate for PPIAS and GPIAS studies, unless longer acclimatization sessions are performed to achieve efficient gap processing. Instead, C57BL/6 or C57BL/6 × 129sv mixed mice (typical of most mouse mutant models available) would be most appropriate at an age when PPIAS/GPIAS responses are maximized and hearing deficits not yet detectable (between 3 and 4 months of age). Longenecker and Galazyuk (2012) also improved gap detection by decreasing the startle stimulus intensity to non-saturating levels (around 105 dB SPL), which has not been implemented in the current experiment. The differences we observe may also be due to differences in sound quality. With the exception of the initial study by Turner and Parrish (2008); Hickox and Liberman (2014), who describe a 48 dB/octave roll-off in their sound filtering like the one used in the present report, none of the other studies that used GPIAS describe the frequency filtering slope of their narrowband noises. Typically, when the shape of the filter is too narrow (e.g., when the slope of the filter is steep), perceptual artifacts are generated and could interfere with the ability of tinnitus to disrupt GPIAS. These artifacts decrease when using a 48 dB/octave roll-off, and could contribute to improved “detectability” of tinnitus. How the contour of the narrowband filtering affects the ability of tinnitus to interfere with GPIAS remains to be addressed. We believe that the appropriate selection of background strain, startle impulse level and ISI can provide robust PPI and gap detection responses when combined with adequate acclimatization sessions. Such parameters should be taken into account when testing gap detection for the assessment of tinnitus in humans.

The identification of GPIAS deficits in the 32-kHz frequency region when using salicylate in GLAST KO mice contrasts with previous research. A recent review article by Galazyuk and Hébert (2015) summarizes the results obtained with different tinnitus models using GPIAS. It appears that the generation of tinnitus by salicylate varies in terms of frequency depending on species and strains (i.e., Wistar rats get broader-range tinnitus, Brown Norways display tinnitus at 10, 12 and 24 kHz, and Sprague Dawleys at 16 kHz). The overall conclusion is that tinnitus does not seem to be focal and its frequency depends on the strain used. It is possible that we may have missed some frequencies in our screening that would have been otherwise revealed using lower carrier sound intensities and a greater number of animals throughout the procedure. Conversely, the protocols using GPIAS to assess tinnitus are very diverse and none of the salicylate studies (with the exception of the article by Turner and Parrish, 2008) have tested potential GPIAS deficits at 32 kHz (Galazyuk and Hébert, 2015). It is thus hard to determine whether our findings are specific to our model, or whether salicylate-induced tinnitus typically triggers high-frequency tinnitus, or simply whether the deficits in GPIAS are truly triggered by tinnitus *per se* and do not result from confounding effects of hearing loss. The control experiments we have performed rule out the potential bias of high-frequency hearing loss in the disrupted GPIAS observed in GLAST KO mice since: (i) auditory thresholds were equally affected by salicylate in WT and KO mice at all frequencies; (ii) salicylate did not affect GPIAS using a BBN carrier sound; (iii) basal auditory thresholds were normal and equivalent in both WT and KO mice at the time of the test; and (iv) salicylate treatment did not disrupt PPI in either WT or KO mice at 32 khZ, which otherwise would have been altered if hearing loss had contributed to the lower GPIAS at 32 kHz found in GLAST KO mice treated with salicylate. However, we do not rule out the possibility that in spite of the significant changes in GPIAS (in either genotype), the lack of changes in PPI upon salicylate administration could arise from salicylate-induced loudness recruitment, which may maintain suprathreshold PPI behavior.

Our study proposes the first gene potentially involved in tinnitus. GLASTs are mainly present in astrocytes in the central nervous system, buffering the excess of glutamate released at the synaptic cleft, converting it into glutamine, which is then transported back to pre-synaptic terminals to be recycled to glutamate. Two major glutamate transporters, namely GLAST (predominant in the cortex and hippocampus) and the glial GLutamate Transporter GLT1 (predominant in the cerebellum), are responsible for more than 80% of the glutamate uptake in the brain. Loss of glutamate transporters with subsequently uncontrolled extracellular glutamate levels has been associated with neurotoxic effects during seizures, amyotrophic lateral sclerosis, epilepsy and now, possibly, tinnitus. Although species comparison in gene expression levels is difficult to evaluate, given the broad changes in endogenous normalizing components (e.g., ubiquitously expressed genes), it appears likely that the resistance of mice to auditory insults could result, at least in part, from the higher expression of cochlear GLAST. On the other hand, a low abundance of cochlear GLAST would predict higher sensitivity to noise and drug-induced tinnitus. Consistent with this notion, we found that GLAST mRNA abundance was greater in CBA mouse cochleae than in those from CD-1 mice (CBA: 1.02 ± 0.06 relative expression level; CD-1: 0.76 ± 0.04; *p* = 0.008 by an unpaired two-tailed *t*-test, *n* = 5 per strain), which correlates with these strains’ known auditory sensitivity and development of age-related hearing loss. In this regard, Shimizu et al. (2005) found that GLAST KO mice are more vulnerable to kanamycin ototoxicity. Ongoing data collection in our laboratory indicates that this is also the case for cisplatin (unpublished observations). In humans, whose susceptibility to ototoxic medications and noise varies from one individual to another, it remains unknown how cochlear GLAST levels correlate with tinnitus predisposition. A recent study failed to detect GLAST protein in support cells from the human cochlea (Ahmed et al., 2013), suggesting that the human cochlea expresses very low levels in GLAST at the afferent synapse. As a consequence, we predict that humans would show less glutamate-buffering capacity and thus greater vulnerability to noise and ototoxic medications. Genetic analyses of the human homolog of GLAST (*EAAT1*) in subjects with and without tinnitus could bring new knowledge about the mechanisms underlying the vulnerability and resilience to tinnitus.

It is rather widely accepted that peripherally generated tinnitus arises from a lack of cochlear output, rather than an increase in cochlear output. There are clear cochlear differences between salicylate and noise insults whereby spontaneous activity of the auditory nerve (AN) is increased after salicylate but decreased after noise (Eggermont and Roberts, 2004). However, most measures in noise-traumatized animals are performed after noise exposure and not *during* noise exposure. Thus, we would predict an early phase of increased AN activity during noise exposure (Searchfield et al., 2004) and a decrease in AN activity resulting from permanent synaptic damage. In contrast to noise, measures on salicylate-treated animals are performed shortly after the administration of salicylate—presumably when salicylate bioavailability peaks in the cochlea. Although there are to our knowledge no studies that report salicylate bioavailability in the cochlea after intraperitoneal injections, a peak is observed in other tissues after 30–60 min, with blood clearance after 8 h (Sturman et al., 1968). As salicylate potentiates glutamate-evoked responses in primary afferent neurons (Ruel et al., 2008), and lack of GLAST results in increased excess glutamate at the IHC-cochlear nerve synapse (Hakuba et al., 2000; Glowatzki et al., 2006), the overall cochlear output in GLAST KO mice would be expected to increase before salicylate is cleared out. Whether repeated administration of salicylate would cause permanent damage is unclear, but a recent rat model of chronic salicylate-administration leads tinnitus (Yi et al., 2016) and could prove useful to investigate whether more permanent damages would occur at the synapse, and reconcile salicylate-induced tinnitus with the decreased cochlear output theory.

The tinnitus effects observed here could also underlie central actions of salicylate and/or loss of GLAST function in the brain. Indeed, systemic administration of salicylate could activate non-auditory structures (Stolzberg et al., 2012; Chen et al., 2015) translating into broader central impacts than those happening after noise exposure (Holt et al., 2010). However, GLAST is predominant in the cortex and the hippocampus, and whether it is present in structures of the auditory pathway is unknown. The higher baseline startle amplitudes observed in GLAST KO mice could reflect hyperacusis. These findings are also consistent with the known higher anxiety levels in this model (Karlsson et al., 2009): increased locomotor activity in the open field, decreased sociability and social novelty preference, and poor nesting behavior. In addition, (Karlsson et al., 2009) found that pairwise discrimination learning is also affected in GLAST KO mice. The startle responses we obtain are however inconsistent with Karlsson et al.’s (2009) findings, which were not controlled—as we did with ABR—for hearing levels (as acknowledged by the authors), known to be affected at 6 months of age in GLAST KO mice (Hakuba et al., 2000). Importantly, unlike the auditory field, which uses startle response as an indicator of hearing abilities, all biological psychiatry textbooks mention the use of the startle response as a gauge of fear or anxiety. For instance, people with post-traumatic stress disorder have greater startle responses (Grillon et al., 1998). After an animal has learned to associate a specific stimulus with fear, such as light being paired with shocks, greater startle response is observed after presenting a fear signal just before the startle stimulus. Conversely, signals associated with pleasure decrease the startle amplitude response (Schmid et al., 1995). Fear or pleasure signals fail to modulate the startle response in amygdala-lesioned animals, showing that the amygdala is involved in the startle response system (Hitchcock and Davis, 1991). We thus believe that the increased startle response seen in GLAST KO mice in the presence of salicylate is an indicator of amygdala-mediated effects. The recent evidence linking tinnitus with the amygdala suggests that central-GLAST deficiency in the amygdala could contribute to the increased tinnitus severity observed in GLAST KO mice. Nonetheless, Karlsson et al. (2009) also found that PPI was not affected in GLAST KO mice and hence reasoned that these mice display *some* of the multiple and complex symptoms belonging to schizophrenia (e.g., GLAST KO mice would belong to a subgroup of schizophrenia of lesser severity). Salicylate, which is known to be anxiogenic in high doses (Puel and Guitton, 2007; Guitton, 2009), exacerbated this anxiety effect by increasing startle amplitude responses in KO animals (Figure 3A), something that we were able to qualitatively observe when handling the animals. It is thus possible that the higher basal anxiety levels of GLAST KO mice facilitated the tinnitus-inducing effects of salicylate, thereby increasing tinnitus intensity. Specific deletion of GLAST either in the brain or in the ear should enable discrimination of the contribution of all of these factors.

## Conclusion

Our results suggest a potential role for GLAST in the vulnerability to salicylate-induced tinnitus. Given the magnitude of the disruption in gap detection observed in GLAST KO mice treated with salicylate, we propose that GLAST deficiency may serve as a useful model to distinguish more subtle, yet unidentified mechanisms on how tinnitus is triggered and maintained. Finally, optimizing parameters in gap detection in humans may uncover a potential use of this technology in the objective diagnosis of tinnitus (Galazyuk and Hébert, 2015).

## Author Contributions

HY, KVP, CH, BF and CRC carried out the experiments; HY, KVP and CRC analyzed the results; CRC designed and directed the research; BC, SS and CRC discussed the results and wrote the manuscript; all authors reviewed the manuscript.

## Funding

CRC was a recipient of postdoctoral fellowships from the Swiss National Science Foundation (SNF; n° PBGEP3-125837), the Schweizerischen Stiftung für medizinisch-biologische Stipendien (SSMBS; n° PASMP3-136979), the Nicholson Fund and the Wenner Gren Foundation, and has received funding from Vetenskapsrådet, Lars Hiertas Minne, Magnus Bergvalls Stiftelserna, Loo och Hans Ostermans, Tysta Skolan, Karolinska Institutet and an independent research program funded under the Biomedicine and Molecular Biosciences European Cooperation in Science and Technology (COST) Action framework (TINNET BM1306). BC received funding from Karolinska Institutet, Vetenskapsrådet, Tysta Skola, Hörselskadades Riksförbund, FORTE and AFA Försäkring. SS is funded by the American Federation for Aging Research, by National Institutes of Health grant DC011840, and by a Claude D. Pepper Older Americans Independence Center Junior Scholar Award.

## Conflict of Interest Statement

CRC received consulting fees from Sensorion Pharmaceuticals.
